# Hybridizing Mg–Fe Layered Double Hydroxide with Pectin Natural Polymer for Organic Ligand-Responsive Phosphate Release: An Innovative Controlled-Release Phosphorus Fertilizer

**DOI:** 10.1021/acs.jafc.4c12454

**Published:** 2025-03-12

**Authors:** Wen-Hui Li, Liang-Ching Hsu, Han-Yu Chen, Yi-Chun Chen, Heng Yi Teah, Yu-Yu Kung, Yu-Min Tzou, Yu-Ting Liu

**Affiliations:** † Department of Soil and Environmental Sciences, National Chung Hsing University, 145 Xingda Rd., Taichung 40227, Taiwan; ‡ Department of Forestry, National Chung Hsing University, 145 Xingda Rd., Taichung 40227, Taiwan; § Presidential Endowed Chair for Platinum Society, The University of Tokyo, 7-3-1, Hongo, Bunkyo-ku, Tokyo 113-8656, Japan; ∥ Innovation and Development Center of Sustainable Agriculture, National Chung Hsing University, 145 Xingda Rd., Taichung 40227, Taiwan

**Keywords:** Mg−Fe LDH, pectin, phosphate sorption and release, organic ligands responsiveness

## Abstract

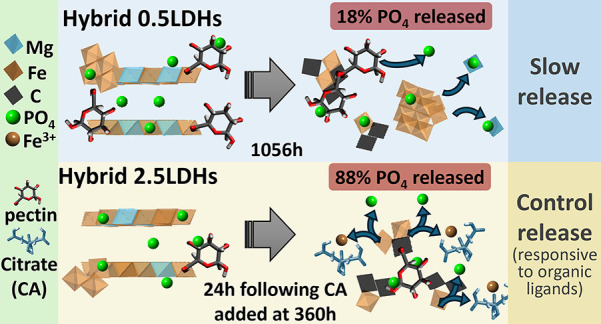

Phosphorus (P) is vital for plant growth, but its agricultural use is limited by soil fixation and environmental loss. This study developed an organic ligand-responsive phosphate release system by hybridizing magnesium–iron-layered double hydroxides (Mg–Fe LDH) with pectin from apple and citrus (pectin-A/C). Structural properties and phosphate (PO_4_) release of LDH hybrids with different concentrations of metal precursors (0.5LDH-A/C, 2.5LDH-A/C) were evaluated. All hybrids exhibited higher PO_4_ sorption than pristine Mg–Fe LDH, with 2.5LDH-A reaching 118.2 mg g^–1^. Phosphate release kinetics showed that 0.5LDH-A/C provided slow release up to 1056 h, while 2.5LDH-A/C released 87.7% PO_4_ with 4 mM citrate, responding to organic ligands. Synchrotron spectroscopy revealed that Fe substitution in LDH layers and Fe(III)-P species was the key influencing PO_4_ release. The slow-release behavior of 0.5LDH-A/C and the ligand responsiveness of 2.5LDH-A/C highlight their potential to enhance sustainable agriculture by improving fertilizer efficiency, ensuring food security, and minimizing environmental impact.

## 1 Introduction

Phosphorus (P) is critical for plant growth; however, P rock is a finite resource, anticipated to deplete within 50 to 100 years.^1^ Agriculture consumes 80–90% of extracted P, but merely 5–30% of applied P is assimilated by plants,^2^ with the remainder lost via soil fixation and runoff. The biogeochemical disruption of P has surpassed planetary boundaries,^3^ and with global food demand projected to rise by 30–62% by 2050,^4^ efficient fertilizer use is crucial.

Recent studies have explored improving P use efficiency through slow-release materials, including recycled P precipitates,^5^ graphene-related materials,^6^ metal–organic frameworks,^7^ and adapted biochar.^8^ Slow-release fertilizers (SRFs) extend nutrient release over time, while controlled-release fertilizers (CRFs) precisely match nutrient release with plant growth stages.^9^ Among promising materials, layered double hydroxides (LDHs) stand out due to their tunable structure, pH-dependent stability, and capacity for anion exchange. LDHs, composed of di- and trivalent metal cations,^10,11^ can intercalate phosphate (PO_4_). The PO_4_ is used in our article as a general representation of phosphate ions, which can exist in various forms (PO_4_^3–^, HPO_4_^2–^, or H_2_PO_4_^–^) depending on the pH. However, PO_4_ sorption on LDH surfaces or impurities formed during preparation (amorphous metal hydroxides) may dominate PO_4_ retention mechanisms,^12^ making ion exchange, desorption, and structural weathering key for PO_4_ release near plant roots. Therefore, the weathering and biodegradability of LDHs over time fulfill the prerequisites for slow release and stimuli-responsive (i.e., controlled release) P fertilizers.

Traditional CRFs are often coated with synthetic polymers that have hydrophobic characteristics to create a diffusion wall or barrier.^13,14^ The most popular coating materials are petroleum-derived synthetic polymers, which may generate toxic byproducts upon degradation. Here, the hybridization of Mg–Fe LDH with pectin, a biodegradable, nontoxic, and natural polymer from apple and citrus,^15^ was investigated as a more sustainable alternative. Pectin is classified by the degree of esterification (DE) into high and low methoxyl pectin (DE ≥ and <50%).^16^ Low methoxyl pectin has more free carboxylic acid groups, while high methoxyl pectin has fewer. When LDH grafts with polymers, the weakened hydrogen bonding and electrostatic interactions between LDH layers promote exfoliation, reducing stacking.^17^ This study hypothesized that varying DE levels may impact the structure and PO_4_ release profile of LDH-pectin hybrids, which may increase the susceptibility to environmental weathering.

Plants under P starvation exude organic acids and ligands like citrate, oxalate, and malate, which trigger responsive PO_4_ release through ligand-promoted Fe complexation.^18^ In the rhizosphere, organic ligand concentrations can exceed 5 mM.^19^ This study aimed to develop CRFs responsive to organic ligands by hybridizing Mg–Fe LDH with high and low DE pectin. It investigated PO_4_ release mechanisms and structural characteristics of the hybrids, using synchrotron-based techniques to assess PO_4_ bonding and Fe coordination. These insights will inform the design of more efficient, ecofriendly P fertilizers, contributing to sustainable agriculture, preserving P reserves, and supporting global food security.

## 2 Materials and Methods

### 2.1 Hybridization between Mg–Fe LDH and Pectin

The Mg–Fe LDHs were hybridized with pectin derived from apple pomace (pectin-A, Sigma-Aldrich, 50–75% DE, Lot # BCCD1493) and citrus peels (pectin-C, Sigma-Aldrich, DE < 26%, Lot # SLCD2368) by means of in situ coprecipitation. Wherein, a 10 mL mixture comprising 1.0 and 5.0 M total Mg [Mg(NO_3_)_2_·6H_2_O] (Merck, 99%, Lot # A1846153 223) and Fe [Fe(NO_3_)_3_·9H_2_O] (Sigma-Aldrich, ≥ 98%, Lot # MKCV3716), with 3:1 molar ratio of Mg:Fe was stirred with 10 mL of 1% (weight/volume) pectin-A or pectin-C suspensions. Pectin-A and pectin-C suspensions were produced via stirring 1 g of apple pectin and citrus peel pectin (MERCK) into degassed and deionized water, adjusting to pH 9.0–10.0 with 1.0 M NaOH, and bringing the total volume to 100 mL. Hereafter, these samples were designated as 0.5/2.5LDH-A/C according to the total Mg and Fe concentration (0.5 and 2.5 M) and the polymer form. Each mixture was then dripped into 50 mL of 1.5 M NaOH, maintaining the pH at 14.0 ± 0.5. After half hour, samples were centrifuged at 9000*g* for 20 min to collect solids, which were rinsed five times with deionized water to eliminate residual ions and then freeze-dried.^20^

### 2.2 Phosphate Sorption and Release of Mg–Fe LDH Hybridized with Pectin-A/C

#### 2.2.1 Sorption Isotherms

Sorption isotherms of PO_4_ on hybrid samples were conducted at pH 5.0 using 10 mM NaNO_3_ as the background electrolyte. Aliquots of 100 mM KH_2_PO_4_ were added into hybrid LDH suspensions to achieve a final ratio of 1 g L^–1^. The pH was maintained at 5.0 using 10 mM HNO_3_ or 10 mM NaOH. Suspensions were shaken for 24 h at 25 °C and then centrifuged at 9000*g* for 20 min. The supernatant was filtered across 0.2 μm membranes. Dissolved PO_4_ was determined colorimetrically via the molybdate method.^21^ Solids were lyophilized for subsequent testing. Sorption results were modeled by means of the Langmuir equation.^22^ Please see Table S1 for the equation details.

#### 2.2.2 Release Kinetics

Solids with the highest observed PO_4_ sorption capacity collected upon sorption isotherms were employed to determine the release behavior. This experiment was performed under 1 g L^–1^ proportion with 3.0 mM NaHCO_3_ at pH 5.5 to mimic plant root environments.^23^ Suspensions were shaken at 25 °C for 1056 h. At predetermined time intervals, aliquots of the suspensions were collected and filtered across 0.2 μm membranes. Dissolved PO_4_ content was determined colorimetrically, while solids were lyophilized for subsequent testing. Results were modeled by the Korsmeyer–Peppas equation.^24^ Please see Table S2 for the equation details.

#### 2.2.3 Phosphate Release with Organic Ligand Addition

Two sets of incubations were performed to test the effect of organic ligands on the PO_4_ release. Both experiments were conducted by using solid samples with the highest observed PO_4_ sorption capacity. Release of PO_4_ was carried out under 1 g L^–1^ proportion, with 3.0 mM NaHCO_3_ at pH 5.5. First, hybrid LDHs with the lowest PO_4_ release efficiency determined via the release kinetics were selected to test the effects of types and concentrations of organic ligands on PO_4_ release. Citrate [HOC(COOH)(CH_2_COOH)_2_ · H_2_O], oxalate (HO_2_CCO_2_H), and malate [HO_2_CCH_2_CH(OH)CO_2_H] were added at concentrations of 1.0, 2.0, and 4.0 mM at the start of PO_4_ release, and the incubations were conducted for 48 h. Next, the organic ligand with the greatest impact on PO_4_ release was added to individual LDH suspensions at concentrations of 1.0, 2.0, and 4.0 mM. This was done at two different time points: simultaneously with the start of the PO_4_ release and after 360 h of release. The incubations were conducted for 24 h. After incubation, suspensions were centrifuged, and supernatants were filtered. Dissolved PO_4_ in the filtrates was then determined by using a colorimetric method. The flowchart of the experimental procedures, including the hybridization of Mg–Fe LDH with pectin-A/C, PO_4_ sorption on the hybrid LDH, and PO_4_ release from the hybrid LDH with and without organic ligand addition, was presented in Figure S1 of the Supporting Information to enhance clarity and facilitate understanding of the workflow.

### 2.3 Crystallographic and Morphological Analyses of Mg–Fe LDH Hybridized with Pectin-A/C

The crystallographic analysis for hybrid LDHs was performed using the X-ray diffraction (XRD) (PANalytical X’Pert Pro MRD). The patterns were acquired from 5 to 65° (2θ) at a scanning rate of 1° (2θ) min^–1^ using Cu-Κα radiation (λ = 1.5406 Å).

A field emission scanning electron microscope (FE-SEM, Ultra Plus, ZEISS) was employed to determine the morphology of hybrid LDHs. Samples were mounted by using copper tape, coated with platinum, and placed in an evacuated chamber. The images were then characterized once the chamber reached the required pressure.

### 2.4 Synchrotron-Based Analyses for Mg–Fe LDH Hybridized with Pectin-A/C Collected during PO_4_ Release

#### 2.4.1 Iron K-Edge Extended X-ray Absorption Fine Structure (EXAFS) Spectroscopy

The local structure of Fe octahedra in hybrid LDHs was analyzed by using Fe K-edge EXAFS spectroscopy. Spectra were acquired at Taiwan Light Source (TLS) beamline 17C1 and Taiwan Photon Source (TPS) beamline 44A at the National Synchrotron Radiation Research Center (NSRRC) in Taiwan. Monochromator energy was calibrated to 7112 eV using Fe foil and monitored during data collection. Data were obtained at energies between −200 and +700 eV relative to 7112 eV. A 0.5 eV step was adopted in the near edge section, and a step size of *k* = 0.05 Å^–1^ was used at higher energies.

Repeated scans per sample were integrated, background removed, and standardized in IFEFFIT software (version 1.2.10).^25^ For EXAFS fitting, the FEFF64 was used to generate the Fe–O, Fe–Mg, and Fe–Fe pairs to 3.50 Å separation according to structural developments of two-line ferrihydrite with limited Mg substitution. The single scattering Fe–C pair was derived from the [Fe(C_2_O_4_)_3_]^3–^ structure.^26^ Coordination number (CN), interatomic distance (Δ*R*), and mean-square displacement of interatomic distance (σ^2^) for all EXAFS data were fitted using a set amplitude reduction factor (*S*_0_^2^ = 0.78), based on the first shell analysis of ferrihydrite and goethite.^27^

#### 2.4.2 Phosphorus K-Edge X-ray Absorption Near Edge Structure (XANES) Spectroscopy

The P K-edge XANES spectra were collected at TPS beamline 32A at the NSRRC. Its monochromator energy was calibrated to 2222.3 eV in accordance with the absorption edge of the Zr L_3_-edge. The harmonic symptoms were minimized by detuning up to 50% of flux at 100 eV higher than the P K-edge absorption edge. The data were acquired between 2090 and 2300 eV, with a 0.2 eV step between 2146 and 2161 eV. Self-absorption effects were deemed negligible since no reduced magnitude of the white-line peak was detected with rising PO_4_ concentrations.^27^

For each sample, data were integrated, the background was removed, and the data were standardized in the interface described above for Fe EXAFS analysis. The P species on hybrid LDHs were identified and quantified by linear combination fitting (LCF) over the spectral section from 2141 to 2181 eV with Athena software. The LCF analyses were performed with end member standards of KH_2_PO_4_ (labile-P), phytic acid (organic-P), and PO_4_ sorbed on ferrihydrite [Fe(III)-P]. Refer to Li et al. (2023)^26^ for more information on P K-edge XANES fitting.

## 3 Results

### 3.1 Characteristics of Mg–Fe LDH Hybridized with Pectin-A/C

**Figure 1:**
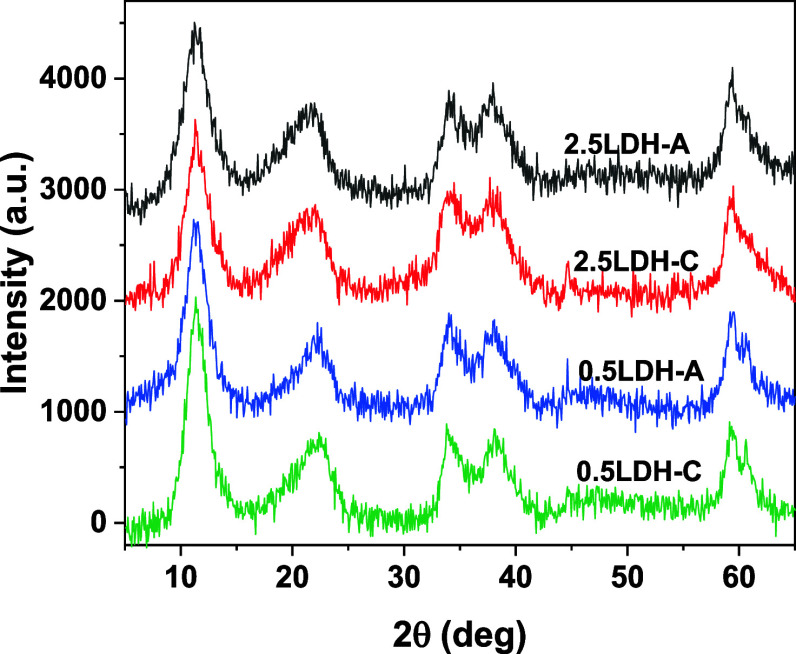
X-ray diffraction patterns of Mg–Fe LDH hybridized with pectin-A/C. Numbers of 2.5 and 1.5 refer to total metal concentrations in LDH precursors. Data were collected by using Cu Kα radiation.

The XRD patterns for all hybrid LDHs showed peaks centered at 2θ values of 11.48, 21.45, 34.16, 37.97, and 59.58° (Figure 1), corresponding to reflections for crystal planes (003), (006), (012), (015), and (110) of Mg–Fe LDH.^28,29^ In contrast to hybrid 2.5LDHs, hybrid 0.5LDHs displayed additional peaks around 60.70°, consistent with the (113) plane for Mg–Fe LDH. This observation suggested that structures of hybrid 0.5LDHs were more crystalline compared with those of hybrid 2.5LDHs. Hence, it can be inferred that the total metal concentrations in LDH precursors played a crucial role in influencing structure development, rather than the type of pectin used.

**Figure 2:**
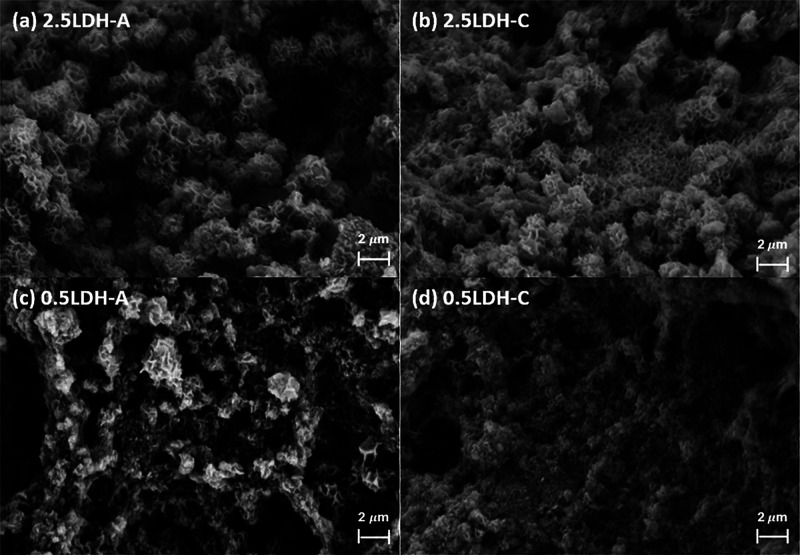
SEM images of Mg–Fe LDH hybridized with pectin-A/C. Numbers of 2.5 and 1.5 refer to total metal concentrations in LDH precursors.

The SEM morphology of hybrid 2.5LDHs revealed the emergence of three-dimensional flowerlike structures with diameters ranging from 1 to 2 μm (Figure 2a,b). This hierarchical architecture mirrors the structural characteristics of pristine Mg–Fe LDH,^26^ facilitating enhanced exposure of particle surfaces. However, such superstructural development was less pronounced in hybrid 0.5LDHs, as evidenced in the random aggregation of particles into clusters with rough shapes, contrasting with the clearly defined microstructural developments observed in hybrid 2.5LDHs (Figure 2c,d).

### 3.2 Phosphate Sorption Isotherms

**Figure 3:**
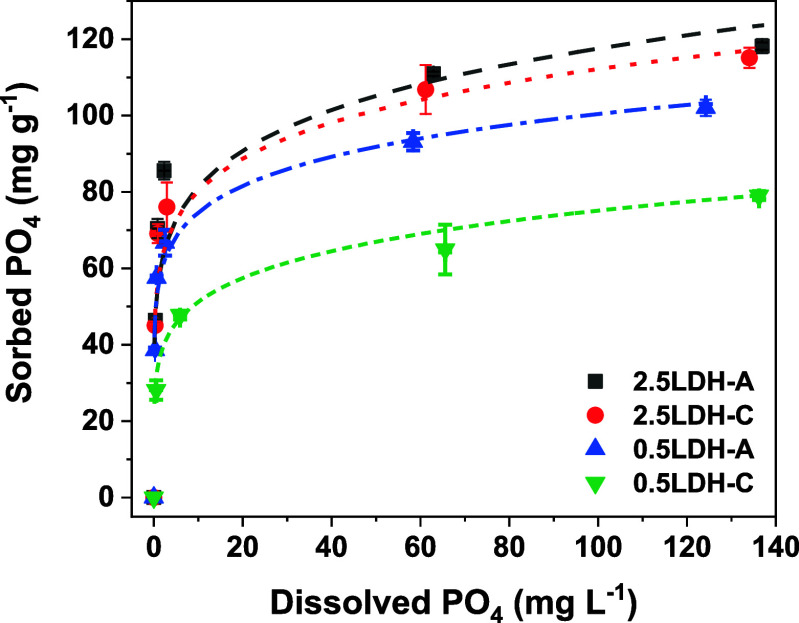
Phosphate sorption isotherms on Mg–Fe LDH hybridized with pectin-A/C. Numbers of 2.5 and 1.5 refer to total metal concentrations in the LDH precursors. The sorption data were fitted using the Langmuir model shown as dashed lines.

Phosphate sorption isotherms on hybrid LDHs were suitably modeled by the Langmuir equation (Figure 3 and Table S1), suggesting nearly monolayer PO_4_ sorption on hybrid LDHs.^22,30^ Regarding modeled maximum sorption capacity, 2.5LDH-A and 2.5LDH-C exhibited comparable amounts of 118.2 and 115.1 mg g^–1^, respectively (Table S1), surpassing that of the hybrid 0.5LDHs (79.1–102.0 mg g^–1^). This sequence of PO_4_ sorption capacities aligned with the structural characteristics of the hybrid LDHs. Wherein, the relatively poor crystalline and microstructural developments of hybrid 2.5LDHs (Figures 1 and 2) demonstrated greater PO_4_ sorption capability.

Although 0.5 M metal concentration in LDH precursors and the addition of pectin-C tended to decrease PO_4_ sorption, PO_4_ sorption capacities of hybrid LDHs here still exceeded that of pristine Mg–Fe LDH [71.8 mg g^–1^, Li et al. (2023)^26^]. For LDHs employing other metal precursors, like Mg–Al LDH and Zn–Al LDH, their PO_4_ sorption capacities varied from 23 to 53 mg g^–1^, contingent upon metal ratios and experiment pH.^22,31^ Moreover, upon hybridizing Mg–Fe LDH with chitosan (CTS) and carboxymethyl cellulose (CMC), the resulting hybrid LDHs exhibited PO_4_ sorption capacities ranging from 58.5 to 66.5 mg g^–1^.^26^ These comprehensive observations essentially suggested that the integration of pectin in Mg–Fe LDH hybridization significantly bolstered its capability for PO_4_ retention.

### 3.3 Phosphate Release Kinetics

**Figure 4:**
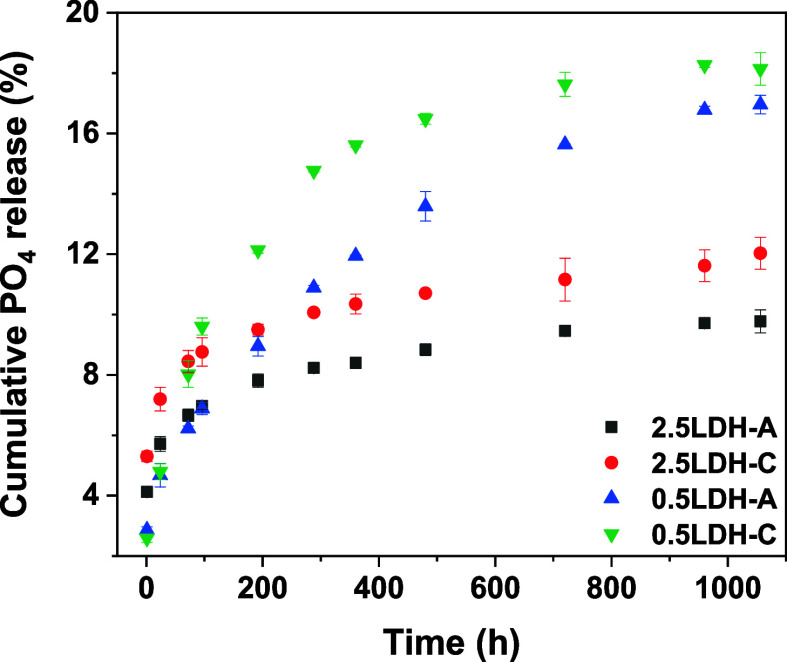
Phosphate release kinetics from Mg–Fe LDH hybridized with pectin-A/C. Numbers of 2.5 and 0.5 refer to total metal concentrations in LDH precursors.

In 24 h, 5.7, 7.2, 4.7, and 4.8% of the sorbed PO_4_ was dissolved from 2.5LDH-A, 2.5LDH-C, 0.5LDH-A, and 0.5LDH-C (Figure 4), suggesting that the PO_4_ release was relatively lower in hybrid LDHs with 0.5 M precursor concentrations. However, by 96 h, the proportions of PO_4_ release had enhanced by an extra 1.3, 1.6, 2.2, and 4.8% for 2.5LDH-A, 2.5LDH-C, 0.5LDH-A, and 0.5LDH-C. Remarkably, the PO_4_ release from 0.5LDH-C doubled between 24 and 96 h. Within 96 to 360 h, the PO_4_ release improved by another 1.4–1.6% for hybrid 2.5LDHs, whereas for hybrid 0.5LDHs, it notably increased by 5.1–6.0%. Reflecting the nearly equilibrated PO_4_ release, hybrid 2.5LDHs only released an additional 1.4–1.7% of sorbed PO_4_ between 360 and 1056 h, while an extra 2.5–5.0% of sorbed PO_4_ was released from hybrid 0.5LDHs. At the end of the incubation, 9.8, 12.0, 17.0, and 18.1% of preloaded PO_4_ was dissolved from 2.5LDH-A, 2.5LDH-C, 0.5LDH-A, and 0.5LDH-C. Based on the fitting results obtained from the Korsmeyer–Peppas equation (Figure S2 and Table S2), the release rate constants of hybrid 2.5LDHs (4.0–5.2 h^–1^) were 1.7 to 2.3 times greater than those of hybrid 0.5LDHs. Considering that a lower rate constant designates slower PO_4_ release, and vice versa, collective results from modeling and experimental data demonstrated that hybrid 0.5LDHs not only released a relatively high proportion of sorbed PO_4_ but also provided protection against rapid PO_4_ release.

Recently, LDH has emerged as a promising material for producing slow-release PO_4_ fertilizers, with Mg–Al LDH commonly used to assess PO_4_ release. Depending on the synthesis conditions of Mg–Al LDH, PO_4_ release in deionized water can range from 60–90% over 53 to 150 min.^31,32^ While equilibrated at pH 8.3 in anion exchange membranes containing 2.0 mM NaHCO_3_, PO_4_ release from Mg–Al LDH can be extended to 650 h.^33^ In our previous study, where Mg–Fe LDH was hybridized with CTS or CMC, the hybrid LDHs with 0.5 M metal precursor (0.5LDH-CTS/CMC) exhibited PO_4_ release that persisted continuously for 2688 h and beyond.^26^ In this study, preloaded PO_4_ amounts on 0.5LDH-A and 0.5LDH-C were 102.0 and 79.0 mg g^–1^, considerably higher than that of 0.5LDH-CTS/CMC (58.5–60.3 mg g^–1^). While only 17.0–18.1% of PO_4_ released from 0.5LDH-A/C at 1056 h, 0.5LDH-CTS/CMC exhibited 20.4–26.5% of PO_4_ release at 1200 h. These comparisons suggested that although 0.5LDH-A/C could lessen the barrier of low PO_4_ loading for practical applications, enhancing PO_4_ release efficiency to align with plant growth time scales and reducing the portion of unreleased PO_4_ in the soil remain significant challenges. According to Everaert et al. (2017),^29^ granulated Mg–Al LDH as SRF did not demonstrate higher agronomic effectiveness compared to the commonly used monoammonium phosphate (MAP) fertilizer. As applied in the form of granule Mg–Al LDH, 74–90% of PO_4_ still remained on the solids upon 100 days of incubation. Although the PO_4_ release efficiency was slightly improved as Mg–Al LDH was applied as a powder, the slow release of PO_4_ could only supplement the dissolved PO_4_ to comparable levels as struvite and MAP did. The PO_4_ release efficiency of our hybrid LDHs has improved compared to that of pristine LDHs, but further enhancing PO_4_ availability is crucial to increase their practical applicability.

### 3.4 Phosphate Release with Organic Ligand Addition

When P is deficient, plants exude organic ligands like citrate, oxalate, and malate around their roots.^18^ Given that up to 5 mM of organic ligands was found as the plant exudation near the rhizosphere,^19^ the organic ligands, including citrate, oxalate, and malate, were added at concentrations from 1 to 4 mM simultaneously during the PO_4_ release. The 2.5LDH-A was selected to test the effects of organic ligands on PO_4_ release over 48 h as its overall PO_4_ release was generally lower than other hybrid LDHs.

**Figure 5:**
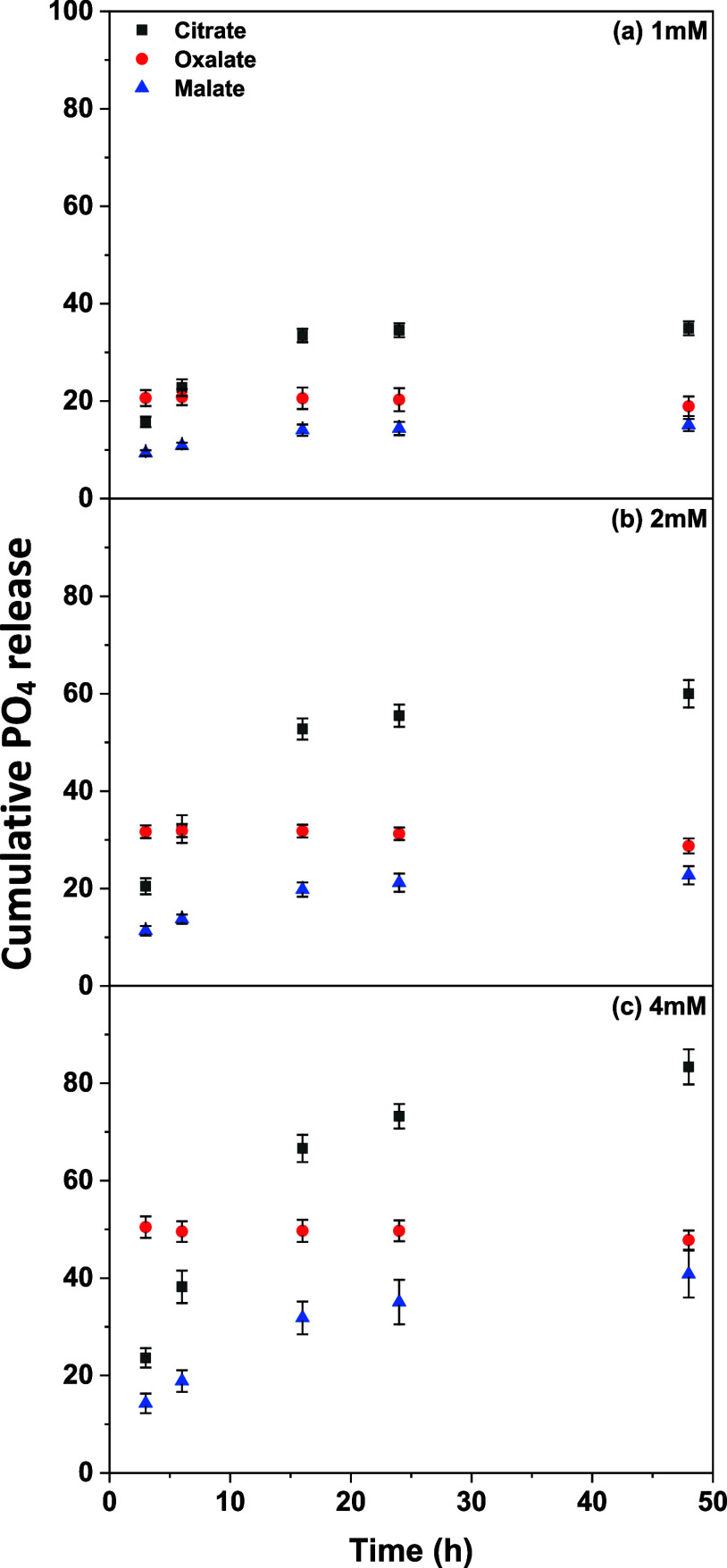
Phosphate release kinetics from Mg–Fe LDH hybridized with pectin-A at a metal precursor concentration of 2.5 M (2.5LDH-A) in the presence of (a) 1, (b) 2, and (c) 4 mM of citrate, oxalate, and malate.

With the addition of 1 mM citrate, there was a significant increase in PO_4_ release, reaching 30.0% at 20 h and stabilizing at a similar range (30.7–31.5%) from 24 to 48 h (Figure 5a). This result suggested that citrate’s strong chelating properties effectively mobilize PO_4_ by disrupting the Mg–Fe LDH structure or competing for sorption sites. As a tricarboxylic acid, citrate has a higher charge density than dicarboxylic acids, such as oxalate and malate, enabling it to form stronger inner-sphere complexes with Fe on the LDH surface. This enhanced complexation ability reduces the availability of binding sites for PO_4_, thereby facilitating its desorption and increasing its mobility in solution.^34,35^ Observations revealed further enhancements in PO_4_ release efficiency at higher citrate concentrations of 2 and 4 mM (Figure 5b,c). At 2 mM, the PO_4_ release sharply increased, reaching 46.9% at 20 h, and maintained levels between 49.9 and 55.5% from 24 to 48 h. The influence on PO_4_ release became even more pronounced with a 4 mM citrate addition, where the PO_4_ release surged to 76.2% by 48 h, with a potential for continual increase over time. Malate, like citrate, showed a concentration-dependent impact on PO_4_ release but was generally less efficient than citrate at comparable concentrations. Within 48 h, PO_4_ releases of 12.2, 18.3, and 30.8% were observed in the presence of 1, 2, and 4 mM malate, respectively. Although oxalate also demonstrated a concentration-dependent effect on PO_4_ release, its pattern diverged notably from that of citrate and malate. At 1 mM, oxalate facilitated PO_4_ release from the onset of incubation, with the release percentages fluctuating between 14.6 and 16.7% over 48 h. This early plateau indicated that oxalate is capable of mobilizing PO_4_ efficiently, yet equilibrium dynamics may restrict further release over time. With 2 mM oxalate, the proportion of PO_4_ released stabilized between 24.5 and 28.7% across all measured time points; yet it substantially surged to 43.1–46.2% over the course of 48-h incubation in the presence of 4 mM oxalate. These findings suggested that oxalate’s impact on PO_4_ release from hybrid LDHs is not significantly time-dependent, underscoring its robust ability to mobilize PO_4_ regardless of incubation duration.

**Figure 6:**
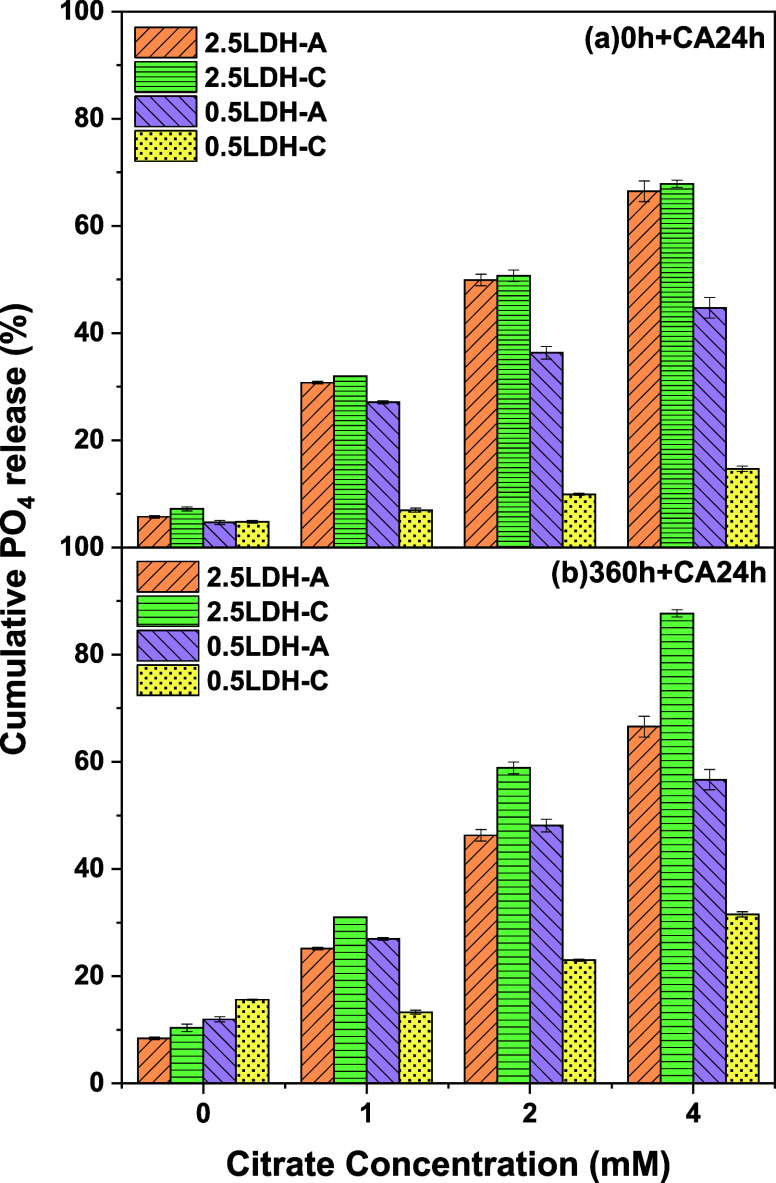
Phosphate release from Mg–Fe LDH hybridized with pectin-A/C observed at 24 h following the addition of 1, 2, and 4 mM citrate. The citrate was introduced either (a) initially or (b) at 360 h into the release incubation. The data labeled as 0 mM for parts a and (b) represent PO_4_ release at 24 and 384 h, respectively, in the absence of citrate addition. Numbers of 2.5 and 0.5 refer to total metal concentrations in LDH precursors.

In summary, citrate proved to be the most effective organic ligand for facilitating the PO_4_ release. Further tests explored the effects of citrate concentration and timing, with citrate added either at the start or 360 h into the incubation. When 1 mM citrate was added at the start of the incubation, PO_4_ release after 24 h from 2.5LDH-A, 2.5LDH-C, 0.5LDH-A, and 0.5LDH-C was 30.7, 31.2, 27.1, and 7.0%, which was 5.4, 4.3, 5.8, and 1.5 times their counterparts without citrate addition (Figure 6a). Clearly, citrate significantly boosted the release of PO_4_ from 2.5LDH-A/C and 0.5LDH-A, though its effect was less pronounced on 0.5LDH-C. As citrate concentrations increased to 2 and 4 mM, the release of PO_4_ also rose sharply. With 4 mM citrate, PO_4_ release from 2.5LDH-A/C and 0.5LDH-A reached 44.7 to 67.9%, a 9.4 to 11.7-fold increase compared to scenarios without citrate, while 0.5LDH-C showed only a 3.1-fold increase.

Given that the release of PO_4_ from 2.5LDH-A/C plateaued around 360 h (Figure 4), citrate was introduced at this point to assess further stimulation. As shown in Figure 6b, adding 1 mM citrate increased PO_4_ release by 3.0 times for 2.5LDH-A/C compared with the levels without citrate at 384 h. However, for 0.5LDH-A/C, 1 mM citrate had less impact or even hindered the PO_4_ release. Notably, the PO_4_ release from 0.5LDH-C was only 90% of the level observed without citrate. Unlike the initial addition, where citrate led to similar PO_4_ release proportion from 2.5LDH-A and 2.5LDH-C, adding citrate at 360 h significantly boosted PO_4_ release from 2.5LDH-C. At 4 mM, PO_4_ release from 2.5LDH-C reached 87.7%, nearly 32% higher than that of 2.5LDH-A. While citrate had a lesser impact on PO_4_ release from 0.5LDH-A/C, their PO_4_ release still increased to 31.5–56.7% with 4 mM citrate (Figure 6b). In cases where 0.5LDH-A/C both released a significant proportion of PO_4_ and prevented its rapid dispersal, they proved to be viable candidates for slow-release PO_4_ fertilizers. In contrast, 2.5LDH-A/C was perceived as more suitable for controlled-release PO_4_ fertilizers, exhibiting a significantly higher responsiveness to citrate exudation than 0.5LDH-A/C. Nevertheless, 2.5LDH-A appeared to reach its PO_4_ release threshold upon citrate addition, as similar rates were observed with both initial and 360-h citrate additions (Figure 6). This suggests that 2.5LDH-A’s slow-release capacity may be compromised after citrate exposure. Regarding 2.5LDH-C, its PO_4_ release not only increased by 2.8 times when citrate was raised from 1 to 4 mM at 360 h but also surpassed that of the counterparts with citrate added initially by up to 1.3 times (Figure 6b). Overall, 2.5LDH-C stands out as a promising option that accommodates citrate-responsive PO_4_ release behaviors.

### 3.5 Iron Local Structures of Mg–Fe LDH Hybridized with Pectin-A/C Collected during PO_4_ Release

Local structural developments surrounding Fe atoms for hybrid LDHs obtained at the start, after 1056 h of PO_4_ release, and 24 h following citrate introduction at 360 h of the release kinetics were analyzed via Fe K-edge EXAFS spectroscopy. Fitting results showed that the first shell coordination of Fe atoms for all samples was oxygen atom with the interatomic distances from 1.97 to 2.01 Å and CN from 4.6 to 6.0 (Table 1 and Figure S3). Although the FeO_6_ octahedron typically coordinates with six oxygen atoms, a structural disorder in Fe domains can reduce the CN.^36^ Varying Fe–O distances may cause destructive interference across wave functions, contributing to this CN reduction.^37^

**Table 1:** Fitting Results of Fe-Edge EXAFS Analysis for Mg–Fe LDH Hybridized with Pectin-A/C Collected at the Start, after 1056 h of PO_4_ Release, and 24 h Following Citrate Introduction at 360 h of the Release Kineticse^a^^,^^b^

path	CN^c^	*R* (Å)^d^	σ^2^ (Å^2^)^e^	path	CN	*R* (Å)	σ^2^ (Å^2^)
2.5LDH-A				0.5LDH-A			
0 h				0 h			
Fe–O	5.1	2.01	0.005	Fe–O	4.6	2.01	0.005
Fe–Mg	1.7	3.12	0.007	Fe–Mg	2.7	3.14	0.006
Fe–Fe	0.5	3.31	0.005	Fe–Fe	1.0	3.54	0.007
1056 h				1056 h			
Fe–O	5.4	1.99	0.009	Fe–O	5.4	1.98	0.009
Fe–C	2.5	2.82	0.013	Fe–C	3.2	2.75	0.012
Fe–Fe	2.2	2.96	0.009	Fe–Fe	3.9	2.96	0.011
Fe–Fe	3.6	3.14	0.011	Fe–Fe	3.6	3.14	0.009
Fe–Fe	1.7	3.39	0.010	Fe–Fe	1.7	3.37	0.009
360 h + CA 24 h				360 h + CA 24 h			
Fe–O	5.5	1.98	0.009	Fe–O	5.9	1.98	0.009
Fe–C	1.2	2.84	0.010	Fe–C	1.5	2.88	0.009
Fe–Fe	2.4	3.03	0.010	Fe–Fe	1.7	3.05	0.010
							
2.5LDH-C				0.5LDH-C			
0 h				0 h			
Fe–O	5.7	2.01	0.006	Fe–O	5.3	2.01	0.005
Fe–Mg	1.7	3.12	0.008	Fe–Mg	3.5	3.12	0.007
Fe–Fe	0.5	3.33	0.005	Fe–Fe	0.6	3.57	0.004
1056 h				1056 h			
Fe–O	5.4	1.98	0.010	Fe–O	5.0	1.98	0.008
Fe–C	2.7	2.75	0.013	Fe–C	3.4	2.77	0.010
Fe–Fe	3.1	2.93	0.012	Fe–Fe	3.4	2.96	0.009
Fe–Fe	4.6	3.11	0.011	Fe–Fe	4.0	3.15	0.010
Fe–Fe	2.0	3.35	0.012	Fe–Fe	2.0	3.39	0.011
360 h + CA 24 h				360 h + CA 24 h			
Fe–O	5.4	1.97	0.009	Fe–O	6.0	1.97	0.009
Fe–C	2.8	2.82	0.012	Fe–C	3.8	2.83	0.010
Fe–Fe	2.6	3.03	0.012	Fe–Fe	2.9	3.03	0.011
				Fe–Mg	0.9	3.10	0.015

a Numbers of 2.5 and 0.5 refer to total metal concentrations in LDH precursors.

b Fitting was done across the *k* range of 2.5 to 10.5 Å^–1^ and the *R* range of 1.0 to 3.5 Å. All samples were fit simultaneously, yielding a normalized sum of squared residuals [*R*-factor = ∑(data-fit)^2^/∑data^2^)] of 0.0071 and 0.0067. Values of other EXAFS model parameters were either fixed or fitted to a common value across all samples as follows: *S*_0_^2^ = 0.78 (fixed amplitude reduction factor based on first-shell fitting of ferrihydrite and goethite); Δ*E* = 0.18 and 0.15 eV (fitted energy shift parameter).

c Coordination number.

d Interatomic distance.

e Mean-square displacements of interatomic distance.

In addition to the Fe–O path, samples obtained at the beginning of PO_4_ release showed higher-shell Fe–Mg and Fe–Fe pairs with separations of 3.12–3.14 Å and 3.31–3.57 Å (Table 1). The presence of Fe–Mg paths indicated that some Mg ions in Mg(OH)_2_ layers were substituted by Fe ions, creating positive charges that allowed PO_4_ anion insertion.^38^ While 2.5LDH-A/C had 1.7 Mg atoms surrounding each Fe atom, the Fe–Mg CN for 0.5LDH-A/C ranged from 2.7 to 3.5, indicating a higher degree of Fe substitution and, consequently, more positive charges on 0.5LDH-A/C. Regarding Fe–Fe pairs, interatomic distances were 3.31–3.33 Å for 2.5LDH-A/C and 3.54–3.57 Å for 0.5LDH-A/C. The shorter distance indicated FeO_6_ octahedra linked by sharing an edge with two OH^–^, while the longer distance suggested double-corner linkages dominated Fe coordination.^39^ However, regardless of the type of linkage, a single linkage may result in a relatively fragile FeO_6_ octahedral structure.

After 1056 h of PO_4_ release, it was noteworthy that no Fe–Mg pairs were detected in the hybrid LDHs. Instead, structural developments for FeO_6_ domains and bonding between FeO_6_ octahedra and carbon were observed in all samples (Table 1). The Fe–C paths, with distances from 2.75 to 2.82 Å, indicated partial hybridization between LDH and natural polymers, likely through the development of Fe-carbonyl assemblages via coordination between FeO_6_ octahedra and carbon monoxide ligands.^40^ Regarding FeO_6_ octahedral linkages, three Fe–Fe pairs with separations from 2.93 to 3.39 Å were found in all samples after 1056 h of PO_4_ release. These structural changes implied the emergence of ferric (oxyhydrox)oxide as it features a shared face with Fe–Fe distances of 2.88–2.90 Å, and shared edges formed by two O^2–^, one O^2–^ and one OH^–^, and two OH^–^, with Fe–Fe separations of ∼2.97 Å, 3.02–3.03 Å, and 3.30–3.35 Å.^39^

Regarding samples obtained after 386 h of PO_4_ release with 4.0 mM citrate added at 360 h, the local Fe structure featured Fe–C and Fe–Fe pairs with separations of 2.82–2.88 Å and 3.03–3.05 Å (Table 1). These paths are attributed to the development of Fe-carbonyl assemblages and shared edges linked by one O^2–^ and one OH^–^. Compared to the ferric (oxyhydrox) oxide structures observed after the PO_4_ release concluded, merely edge-shared FeO_6_ octahedral linkages remained after citrate addition, implying structural disruption due to ligand-promoted Fe dissolution. Beyond these structural developments, 0.5LDH-C displayed two unique features. First, the CN of the Fe–C pair in 0.5LDH-C was 3.8, 1.4–3.2 times higher than that in other citrate-treated samples, yet similar to its counterpart acquired upon 1056 h of PO_4_ release without citrate. Additionally, the Fe–Mg pair with a 3.10 Å separation persisted in 0.5LDH-C despite ligand-promoted dissolution. These attributes suggested a relatively robust structure, indicating that interlayers with Mg substituted with Fe ions were partially maintained in 0.5LDH-C.

### 3.6 Phosphorus Speciation on Mg–Fe LDH Hybridized with Pectin-A/C Collected during PO_4_ Release

**Figure 7:**
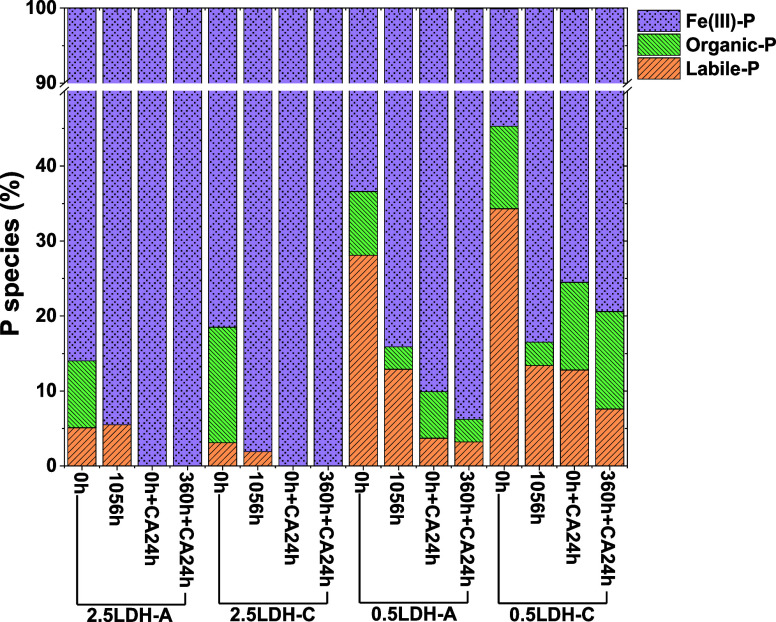
Phosphorus speciation was obtained from P K-edge XANES LCF analysis. Tested samples included Mg–Fe LDH hybridized with pectin-A/C collected at the beginning and after 1056 h of PO_4_ release as well as those with citrate introduced either at the start or after 360 h of the release kinetics and incubated for additional 24 h. Numbers of 2.5 and 0.5 refer to total metal concentrations in LDH precursors. Reference materials used to represent labile-P, organic-P, and Fe(III)-P were KH_2_PO_4_, phytic acid, and PO_4_ sorbed on ferrihydrite, respectively.

Phosphorus species on hybrid LDHs were identified and quantified via LCF for their P K-edge XANES spectra (Figure S4). Results of LCF (Table S3 and Figure 7) showed that all tested samples contained labile-, organic-, and Fe(III)-P. Consistent with Fe local structures in hybrid LDHs, which showed Fe substitution in interlayers and the formation of Fe(III) (oxyhydrox) oxides (Table 1), labile- and Fe(III)-P can be designated as intercalated and Fe(III)-bonded PO_4_. The presence of organic-P suggested an alternative retention mechanism through association between PO_4_ and organic compounds in hybrid LDHs. Since PO_4_ typically bonds to Fe(III) (oxyhydrox)oxides via monodentate or bidentate inner sphere complexes,^12^ this P species is less prone to release compared to labile- and organic-P. Therefore, the total of labile- and organic-P represented readily releasable P. This proportion was 14.0–18.5% on 2.5LDH-A/C at the onset of PO_4_ release but dropped to 1.9–5.5% after 1056 h (Figure 7). Conversely, the total of labile- and organic-P on 0.5LDH-A/C initially reached 36.6–45.3% and, although decreased, remained at 15.9–16.5% after 1056 h. This relatively higher proportion at the end of the release incubation might explain why the release of PO_4_ from 0.5LDH-A/C had not reached a steady state, while that from 2.5LDH-A/C appeared to plateau (Figure 4).

When 4.0 mM citrate was added, either at the start or at 360 h of PO_4_ release, no labile- or organic-P was found on 2.5LDH-A/C (Table S3 and Figure 7). Instead, Fe(III)-P dominated the P species. Despite cumulative PO_4_ release of 66.5–87.7% from 2.5LDH-A/C with 4.0 mM citrate added at the start and 360 h (Figure 6), their initial labile- and organic-P inventory was only 14.0–18.5%. This suggested Fe(III)-P was the primary source of PO_4_ release from 2.5LDH-A/C, making it highly responsive to citrate because of Fe(III)’s susceptibility to ligand-promoted complexation.^41^

For 0.5LDH-A/C, readily releasable P was still present whether citrate was added at the start or at 360 h. With 4.0 mM citrate, 6.2–9.9% of readily releasable P persisted on 0.5LDH-A after 24 or 384 h of PO_4_ release, while 20.6–24.5% remained on 0.5LDH-C. Notably, this proportion on 0.5LDH-C was higher than that on its counterpart collected after 1056 h of PO_4_ release (16.5%, Figure 4). This suggested that although Fe(III)-P on 0.5LDH-A/C underwent ligand-promoted dissolution, intercalated and organic-bound P was still retained, especially on 0.5LDH-C. Such P species distribution aligned with the relatively higher Fe–C CN and the retained layered structure with Fe substitution observed on 0.5LDH-C after PO_4_ release with citrate addition (Table 1). This finding explained the minimal increase in the level of PO_4_ release from 0.5 LDH-C in response to 4.0 mM citrate addition at the start and 360 h of incubation (Figure 6). However, multiple release mechanisms enabled 0.5LDH-C to support both slow and controlled PO_4_ release.

While the labile-P was designated as intercalated PO_4_, there might be a possible overestimation, as Mg-bonded P has a similar P-XANES spectral feature. However, Mg_3_(PO_4_)_2_ precipitation can be excluded as its spectral characteristics were indiscernible in any samples. Additionally, the presence of intercalated PO_4_ contradicted the absence of Fe–Mg paths, which would indicate Fe substitution in hybrid LDHs after 1056 h of PO_4_ release. The likely explication is that EXAFS analysis only captures dominant structural information with a distribution >10%.^42^ Therefore, the lack of Fe–Mg paths might indicate a reducing amount of Fe substitution instead of its utter lack.

## 4 Discussion

The 2.5LDH-A/C and 0.5LDH-A/C samples showed PO_4_ sorption capacities 1.6 and 1.1–1.4 times higher than those of pristine Mg–Fe LDH, indicating that pectin hybridization improved PO_4_ loading for practical applications. After 1056 h of incubation, 9.8, 12.0, 17.0, and 18.1% of preloaded PO_4_ was released from 2.5LDH-A, 2.5LDH-C, 0.5LDH-A, and 0.5LDH-C. Although 0.5LDH-A/C had higher PO_4_ release proportions, their release rate constants were only 0.4–0.6 times those of 2.5LDH-A/C, indicating slower PO_4_ release. These results suggested that 0.5LDH-A/C was better suited as a P SRF, offering protection against rapid PO_4_ release while maintaining agronomic effectiveness.

However, the level of release of PO_4_ from 2.5LDH-A/C significantly increased compared to that of 0.5LDH-A/C with organic ligand addition. Among citrate, oxalate, and malate, citrate showed the strongest impact, with 4 mM citrate triggering 76.2% PO_4_ release from 2.5LDH-A, which was 1.8–2.5 times greater than oxalate and malate. Citrate also showed potential for continuous PO release beyond 48 h, unlike oxalate, which peaked at the start. Citrate was further tested both initially and 360 h after release incubation. Initially, PO_4_ release from 2.5LDH-A and 2.5LDH-C was comparable, but adding 4 mM citrate at 360 h significantly boosted PO_4_ release from 2.5LDH-C to 87.7%, which is 1.3 times that of 2.5LDH-A. In contrast, PO_4_ release from 0.5LDH-A/C with 4 mM citrate added at 360 h was only 31.5–56.7%. These findings suggested that hybrid 2.5LDHs, especially 2.5LDH-C, are promising candidates for P CRF reagents responsive to citrate exudation.

The P K-edge XANES results showed that 0.5LDH-A/C had higher proportions of readily releasable P (36.6–45.3%, including labile- and organic-P) compared to 2.5LDH-A/C at the onset of PO_4_ release, explaining why PO_4_ release from 0.5LDH-A/C had not plateaued, unlike 2.5LDH-A/C after 1056 h. Even after citrate addition, 20.6% of readily releasable P persisted on 0.5LDH-C upon 384 h, whereas only Fe(III)-P was found on 2.5LDH-A/C. These findings aligned with Fe-EXAFS data showing Fe–Mg pairs, indicating Fe substitution in Mg(OH)_2_, present in all samples at the start of PO_4_ release but only on 0.5LDH-C after citrate incubation. Additionally, 0.5LDH-C exhibited a relatively higher Fe–C CN. This robust structural development and sustained Fe substitution made 0.5LDH-C suitable as a P SRF, even with citrate addition. Meanwhile, the dominance of Fe(III)-P on 2.5LDH-A/C, particularly on 2.5LDH-C, ensured that the PO_4_ release is highly responsive to citrate. Taken together, hybridizing Mg–Fe LDH with pectin created both SRF and CRF. The metal concentration in the LDH precursor and the pectin type determined the PO_4_ release behavior. Additionally, ferric (oxyhydrox)oxides left after PO_4_ release could offer additional benefits by providing retention sites for soil organic matter. Ultimately, this approach could support food security and climate change mitigation, in line with the Sustainable Development Goals.

In terms of cost analysis, the production cost of Mg–Fe LDH hybridized with pectin as P fertilizers is estimated at 2.54–2.85 USD/kg, whereas commercial SRF, such as granulated triple superphosphate (TSP), is significantly cheaper at 0.35 USD/kg. However, LDH-based P fertilizers may substantially reduce the environmental cost of excess phosphorus leaching, lowering it to 1062 USD–2667 USD per hectare, compared to 2590 USD–4604 USD per hectare for TSP application (see details in Supporting Information). A comprehensive evaluation of both production and environmental costs highlights hybrid LDH as a more sustainable and cost-effective alternative to conventional SRFs. Despite these advantages, several challenges must be addressed for their widespread adoption. Scaling up production remains a key issue as cost reductions are necessary to compete with conventional P fertilizers. Additionally, the long-term agronomic effectiveness of LDH-based fertilizers under diverse soil and crop conditions requires further validation through field trials and multiseason studies. To enhance the sustainability, feasibility, and scalability of Mg–Fe LDH hybridized with pectin, an integrated approach combining fertilizer effectiveness studies with life cycle assessment is crucial. This will provide a comprehensive evaluation of crop yield, production costs, and environmental impacts, ensuring that LDH-based fertilizers can be optimized for both economic and ecological benefits. Addressing these challenges is essential for the successful commercialization and large-scale implementation of LDH-based P fertilizers.

## Supporting Information Available

Flowchart of experimental procedures; supporting tables and figures for phosphate sorption and release models; EXAFS and XANES analysis of structural and chemical changes; and detailed description of production cost analysis and environmental impact assessment (PDF)
