# Supplementary material for: Hybridizing Mg–Fe Layered Double Hydroxide with Pectin Natural Polymer for Organic Ligand-Responsive Phosphate Release: An Innovative Controlled-Release Phosphorus Fertilizer
Source: J Agric Food Chem. 2025 Mar 12;73(12):7131–9. doi: 10.1021/acs.jafc.4c12454 (PMC11951156; doi:10.1021/acs.jafc.4c12454)
Supplement: Supplementary file 1 — jf4c12454_si_001.pdf [file jf4c12454_si_001.pdf]

Supplementary material

Hybridizing Mg-Fe layered double hydroxide with pectin natural polymer for organic ligand-responsive phosphate release: an innovative controlled-release phosphorus fertilizer

Wen-Hui Li[a#], Liang-Ching Hsu[a#], Han-Yu Chen[a], Yi-Chun Chen[b], Heng Yi Teah[c], Yu-Yu Kung[a], Yu-Min Tzou[a,d], Yu-Ting Liu[a,d*]

[a] Department of Soil and Environmental Sciences, National Chung-Hsing University, 145 Xingda Rd., Taichung 40227, Taiwan.

[b] Department of Forestry, National Chung Hsing University, 145 Xingda Rd., Taichung 40227, Taiwan.

[c] Presidential Endowed Chair for Platinum Society, The University of Tokyo, 7-3-1, Hongo, Bunkyo-ku, Tokyo 113-8656, Japan.

[d] Innovation and Development Center of Sustainable Agriculture, National Chung Hsing University, 145 Xingda Rd., Taichung 40227, Taiwan.

[#]These authors contributed equally
[*]Corresponding author
Yu-Ting Liu
Department of Soil and Environmental Sciences,
National Chung Hsing University,
145 Xingda Rd., Taichung 40227, Taiwan,
E-mail address: yliu@nchu.edu.tw
ORCID: 0000-0003-3738-7125

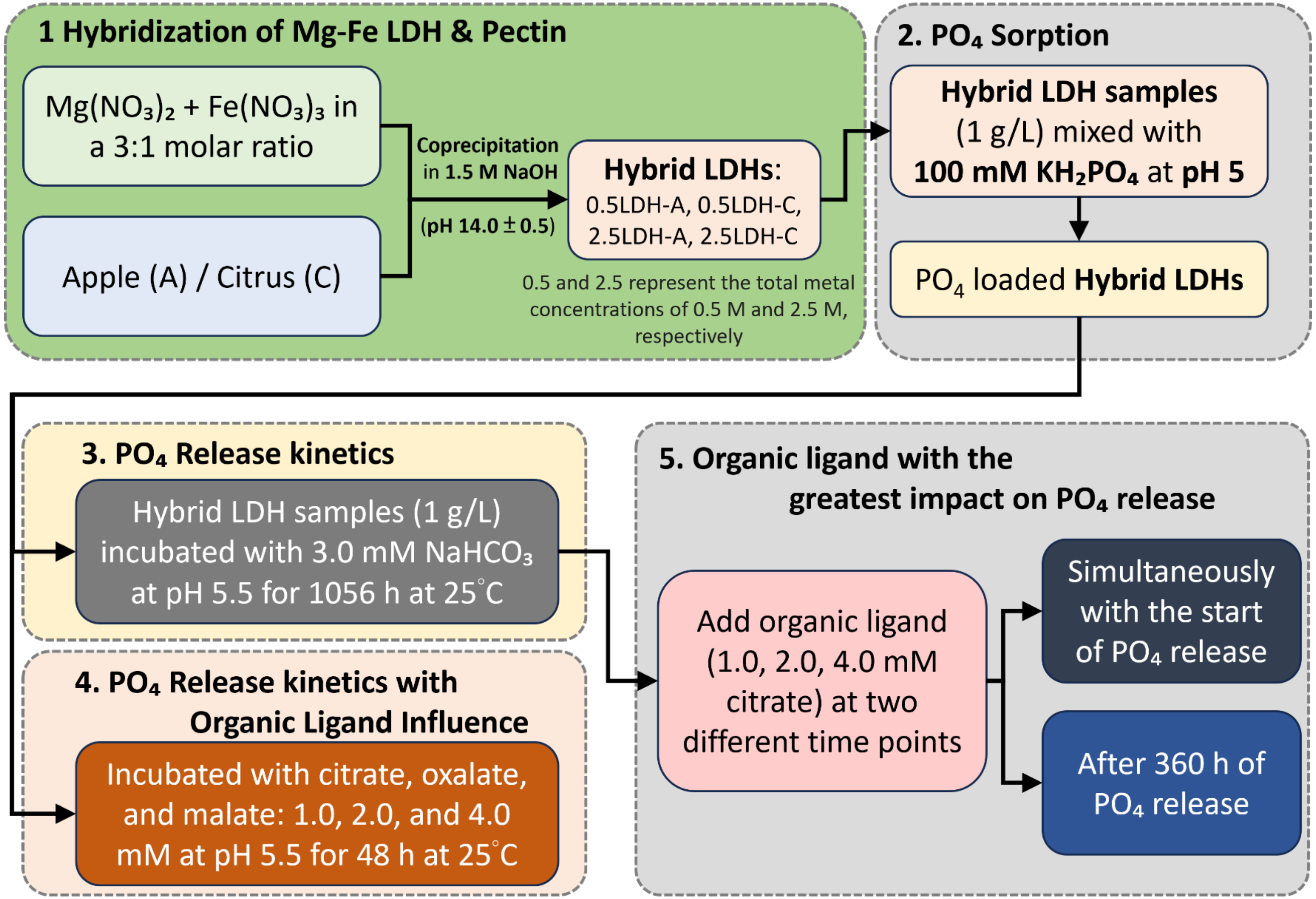

Fig. S1. Flowchart of the experimental procedures, including hybridization between Mg-Fe LDH and pectin-A/C, PO_4 sorption on hybrid LDH, PO_4 release from hybrid LDH with and without organic ligand addition.

Table S1. Fitting parameters obtained from the Langmuir model for PO_4 sorption on Mg-Fe LDH hybridized with pectin-A/C. Numbers of 2.5 and 1.5 refer to total metal concentrations in LDH precursors.

Sample	Q_m[a] (mg g^{-1})	K_L[b] (L g^{-1})	R^2[c]
2.5LDH-A	118.2	2.14	0.951
2.5LDH-C	115.1	2.36	0.932
0.5LDH-A	102.0	2.41	0.901
0.5LDH-C	79.1	0.31	0.995

[a] Q_m is the maximum PO_4 sorption capacity.

[b] K_L means the Langmuir model constant.

[c] R^2 represents the correlation coefficient.

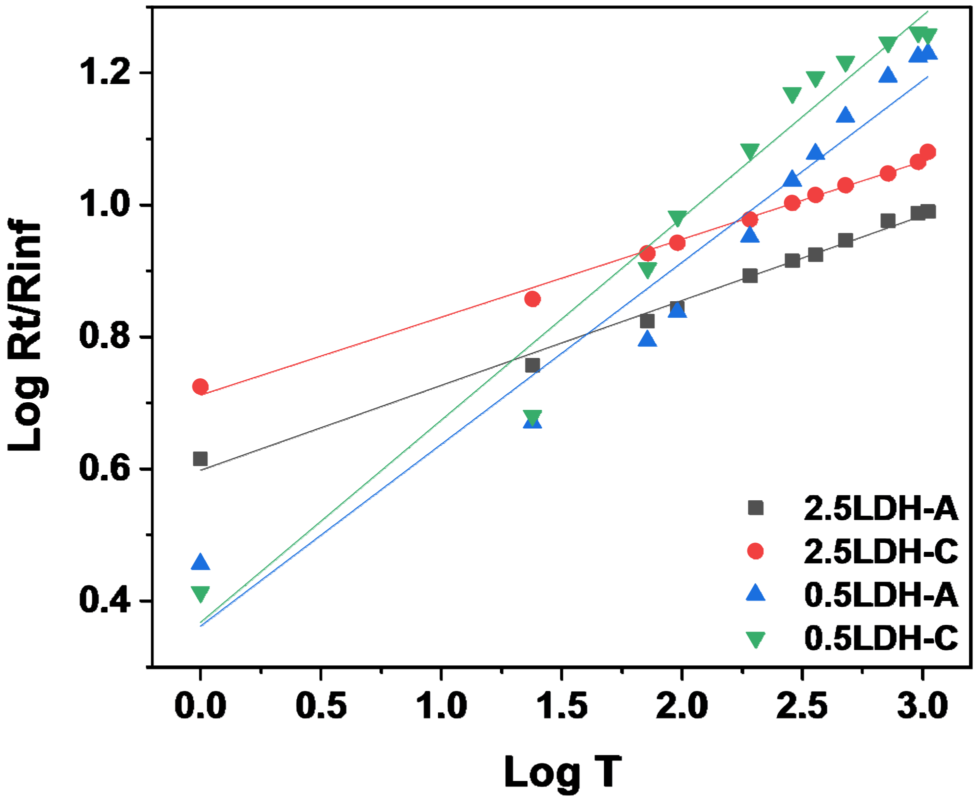

Fig. S2. Fitting results of the Korsmeyer-Peppas model for PO_4 release from Mg-Fe LDH hybridized with pectin-A/C. Numbers of 2.5 and 0.5 refer to total metal concentrations in LDH precursors.

Table S2. Fitting parameters of the Korsmeyer–Peppas model for PO_4 release from Mg-Fe LDH hybridized with pectin-A/C. Numbers of 2.5 and 0.5 refer to total metal concentrations as the LDH precursors.

Sample	Release exponent (n)[a]	Release rate constant (k, h^{-1})	R^2
2.5LDH-A	0.13	4.0	0.991
2.5LDH-C	0.12	5.2	0.994
0.5LDH-A	0.28	2.3	0.947
0.5LDH-C	0.30	2.3	0.971

[a] The release exponent characterizes the release mechanism, where $n \leq 0.45$ denotes a Fickian diffusion transport, and $0.45 < n < 0.87$ signifies a non-Fickian diffusion transport.

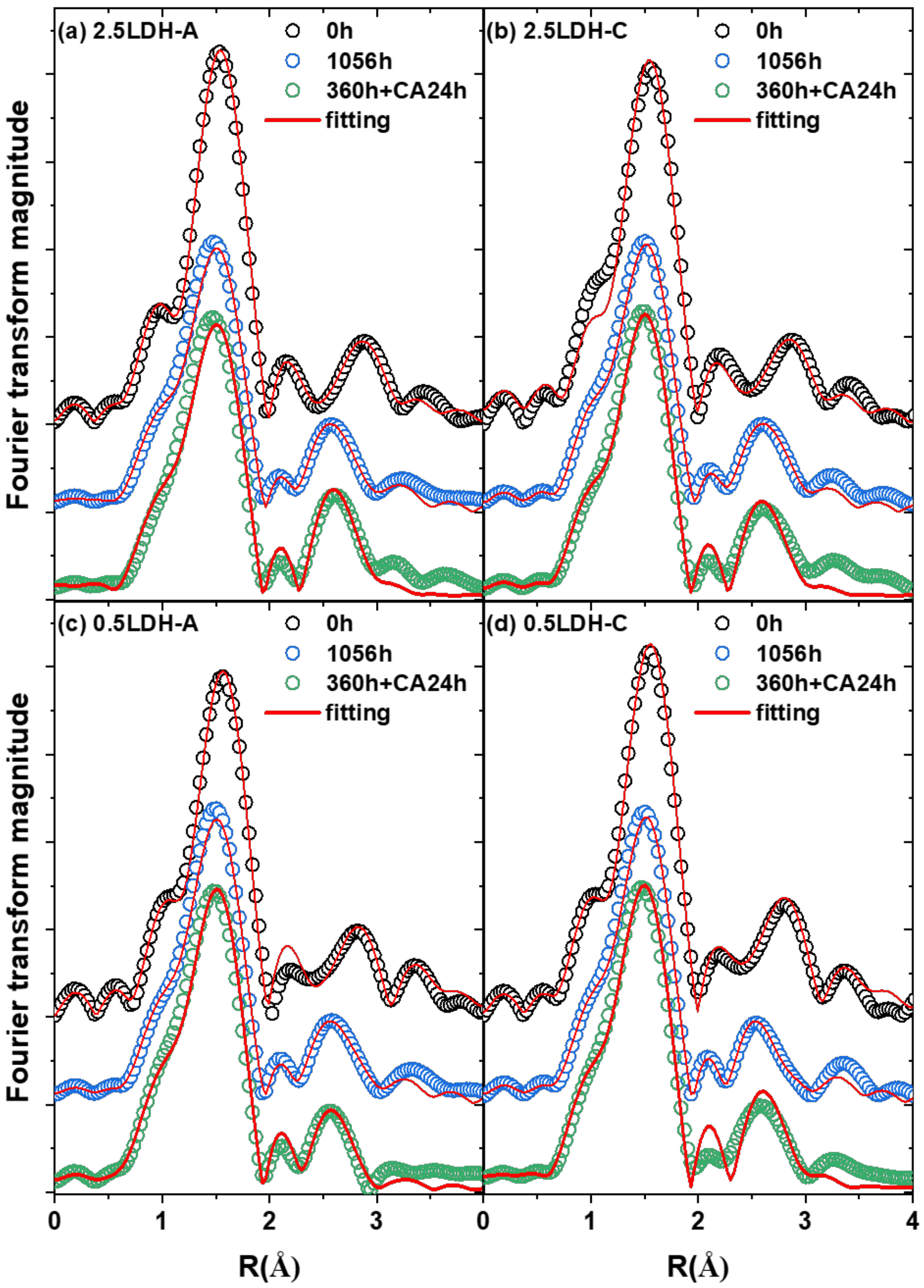

Fig. S3. Fourier-transformed Fe K-edge EXAFS data (open circles) and model fits (solid lines) for Mg-Fe LDH samples hybridized with pectin-A/C. The samples included 2.5 and 0.5 M total metal concentrations in the LDH precursors: (a) 2.5LDH-A, (b) 2.5LDH-C, (c) 0.5LDH-A, and (d) 0.5LDH-C. For each hybrid LDH, samples were collected at the start, after 1056 h of PO_4 release, and 24 h following citrate introduction at 360 h of the release kinetics.

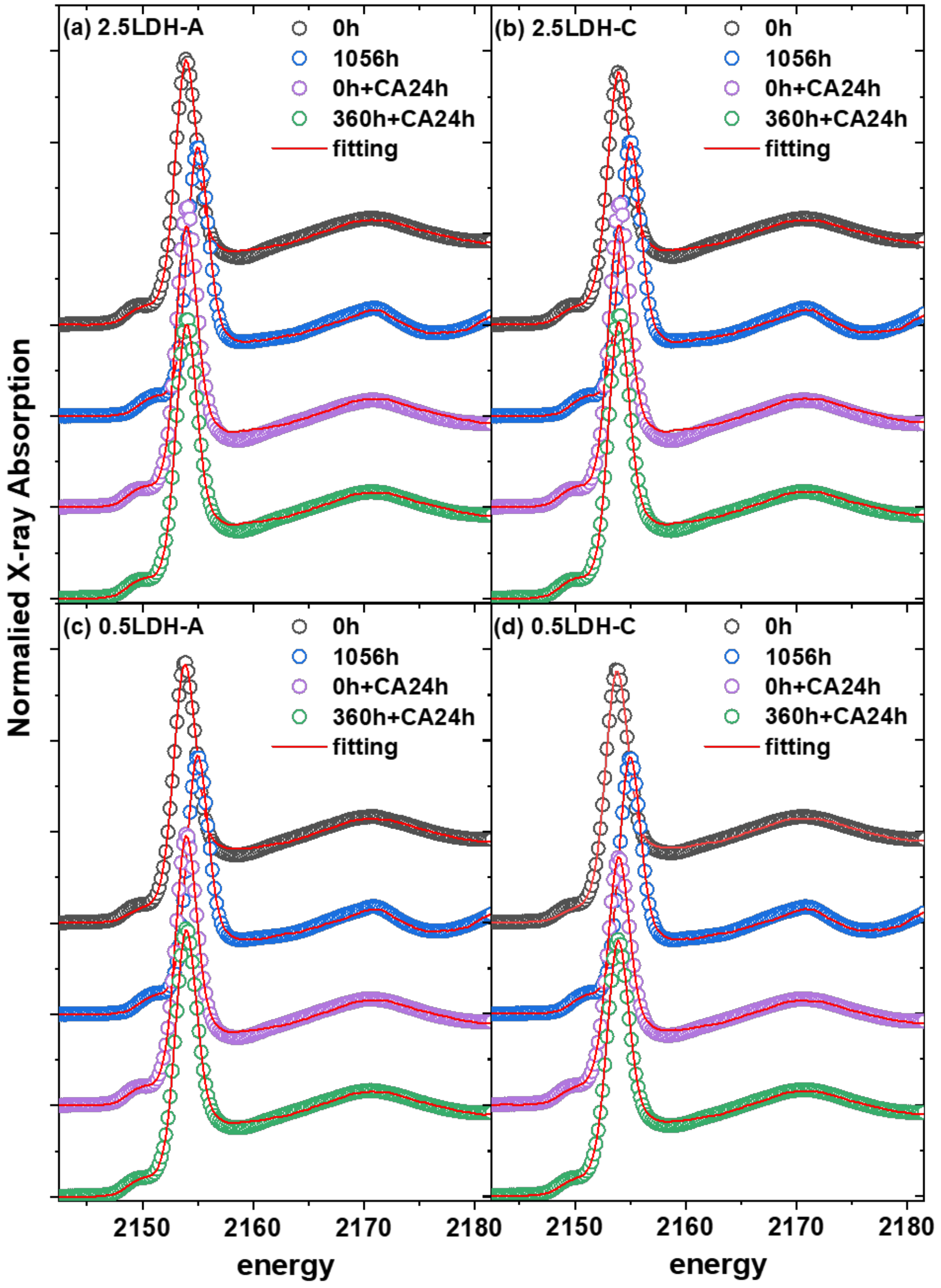

Fig. S4. Phosphorus K-edge XANES data (open circles) and LCF fitting results (solid lines) for Mg-Fe LDH samples hybridized with pectin-A/C. The samples included 2.5 and 0.5 M total metal concentrations in the LDH precursors: (a) 2.5LDH-A, (b) 2.5LDH-C, (c) 0.5LDH-A, and (d) 0.5LDH-C. For each hybrid LDH, samples were collected at the start, after 1056 h of PO_4 release, and 24 h following citrate introduction at the beginning and 360 h of the release kinetics.

Table S3. Results of P K-edge XANES LCF analysis. Tested samples included Mg-Fe LDH hybridized with pectin-A/C collected at the beginning and after 1056 h of PO_4 release, as well as those with citrate introduced either at the start or after 360 h of the release kinetics and incubated for additional 24 h. Numbers of 2.5 and 0.5 refer to total metal concentrations in LDH precursors. [a,b]

Sample		Labile-P (mol %)	Organic-P (mol %)	Fe(III)-P (mol %)	R-factor[c]
2.5LDH-A	0h	5.10 ± 0.6	8.90 ± 0.7	86.1 ± 0.9	0.0013
	1056h	5.50 ± 0.6	-/-	94.5 ± 0.5	0.0006
	0h+CA24h[e]	-/-	-/-	100.0 ± 1.9	0.0014
	360h+CA24h	-/-	-/-	100.0 ± 3.7	0.0049
2.5LDH-C	0h	3.10 ± 4.5	15.4 ± 3.7	81.5 ± 0.9	0.0012
	1056h	1.90 ± 0.4	-/-	98.1 ± 0.4	0.0003
	0h+CA24h	-/-	-/-	100.1 ± 2.1	0.0015
	360h+CA24h	-/-	-/-	100.0 ± 4.2	0.0061
0.5LDH-A	0h	28.1 ± 5.4	8.50 ± 4.4	63.4 ± 1.1	0.0017
	1056h	12.9 ± 4.2	3.00 ± 0.4	84.1 ± 2.1	0.0011
	0h+CA24h	3.70 ± 0.5	6.20 ± 0.6	90.1 ± 0.7	0.0009
	360h+CA24h	3.20 ± 0.6	3.00 ± 0.7	93.7 ± 0.8	0.0010
0.5LDH-C	0h	34.3 ± 4.6	11.0 ± 3.7	54.6 ± 0.9	0.0012
	1056h	13.4 ± 4.4	3.10 ± 0.4	83.5 ± 2.2	0.0012
	0h+CA24h	12.8 ± 3.5	11.7 ± 2.9	75.4 ± 0.7	0.0007
	360h+CA24h	7.60 ± 3.5	13.0 ± 3.3	79.4 ± 0.6	0.0010

[a] Reference materials used to represent labile-P, organic-P, and Fe(III)-P were KH_2PO_4, phytic acid, and PO_4 sorbed on ferrihydrite, respectively.

[b] The weighting factors on each fit were summed to 100 ± 1% and were normalized to 100%.

[c] Normalized sum of the squared residuals of the fit (R-factor = $\sum(\text{data-fit})^2/\sum \text{data}^2$).

Cost analysis of production and environmental impacts: Mg-Fe LDH hybridized with pectin A/C vs. granulated triple superphosphate (commercial slow-release fertilizer)

The production costs of Mg-Fe LDH hybridized with pectin A/C were estimated based on the market prices of industrial-grade raw materials, as listed below:

- $Mg(NO_3)_2 \cdot 6H_2O$: 0.45 USD/kg
- $Fe(NO_3)_3 \cdot 9H_2O$: 0.60 USD/kg
- NaOH: 0.125 USD/kg
- Pectin-Apple: 0.58 USD/kg
- Pectin-Citrus: 0.57 USD/kg

According to the synthesis methods described in Section 2.1: Hybridization between Mg-Fe LDH and pectin, the raw material weights used to synthesize 0.5LDH-A/C and 2.5LDH-A/C, along with the resulting product weights, are as follows:

2.5LDH-raw materials

- $Mg(NO_3)_2 \cdot 6H_2O$: 9.64 g
- $Fe(NO_3)_3 \cdot 9H_2O$: 5.05 g
- NaOH: 3 g
- Pectin A/C: 0.1 g

2.5LDH-products

- 2.5LDH-A: 3.07 g
- 2.5LDH-C: 3.00 g

0.5LDH-raw materials

- $Mg(NO_3)_2 \cdot 6H_2O$: 1.93 g
- $Fe(NO_3)_3 \cdot 9H_2O$: 1.01 g
- NaOH: 3 g
- Pectin A/C: 0.1 g

05LDH-products

- 0.5LDH-A: 0.67 g
- 0.5LDH-C: 0.67 g

Based on these synthesis ratios, the calculated production costs for each product are:

- 2.5LDH-A: 2.54 USD/kg
- 2.5LDH-C: 2.60 USD/kg
- 0.5LDH-A: 2.85 USD/kg
- 0.5LDH-C: 2.84 USD/kg

The production costs of 0.5LDH-A/C and 2.5LDH-A/C range between 2.54–2.85 USD/kg, which is lower than the estimated 3.5 USD/kg for Mg-Al LDH.[1] However, the production costs for both pure and hybrid LDH remain significantly higher than those of commercial slow-release P fertilizers, such as granulated triple superphosphate (TSP), which has a market price of 0.35 USD/kg. For P-deficient soils, the recommended P fertilization rate is 130 mg/kg.[2] Given a soil depth of 10 cm and a bulk density of 1.36 g/cm^3,[3] the total soil mass per hectare is 2,040,000 kg, requiring a total P fertilizer application of 265.2 kg P/ha. This translates to a fertilization cost per hectare of 92.82 USD/ha for TSP and 673.87–926.87 USD/ha for LDH.

To provide a meaningful cost comparison, we also estimated the environmental costs associated with P leaching from LDH-based P fertilizers vs. TSP. After application, TSP exhibits significantly higher P leaching rates, ranging between 21.7–23.3%, whereas LDH demonstrates considerably lower losses, ranging from 8.9–13.5%.[4] The environmental cost associated with excessive P release—factoring in cleanup, recycling, and mitigation efforts—is estimated at 45–74.5 USD/kg of excess P.[5] This results in an environmental cost per hectare of 2,590–4,604 USD for TSP application, whereas LDH substantially reduces this cost to 1,062–2,667 USD/ha. When considering hidden environmental costs, hybrid LDH emerges as a more sustainable and cost-effective alternative in the long run. Its ability to reduce P losses while ensuring efficient nutrient availability highlights its potential as an economically and environmentally viable solution for sustainable agriculture.

(1) Ganem, H. E.; Zohar, I.; Litaor, M. I.; Jonas-Levi, A.; DiSegni, D. M. Phosphorus in Balance: Evaluating the Costs and Benefits of a Circular Economy Approach. **2024**.
(2) Negassa, W.; Leinweber, P. How does the Hedley sequential phosphorus fractionation reflect impacts of land use and management on soil phosphorus: A review. *Journal of Plant Nutrition and Soil Science* **2009**, *172* (3), 305-325.
(3) Keller, T.; Håkansson, I. Estimation of reference bulk density from soil particle size distribution and soil organic matter content. *Geoderma* **2010**, *154* (3-4), 398-406.
(4) Liu, X.; Zhi, Y.; Tian, Y.; Yuan, Y.; Zhong, H. Phosphate-Adsorbed by Concrete-Based Layered Double Hydroxide: A Slow-Release Phosphate Fertilizer. *Polish Journal of Environmental Studies* **2024**, *33* (5).
(5) Sampat, A. M.; Hicks, A.; Ruiz-Mercado, G. J.; Zavala, V. M. Valuing economic impact reductions of nutrient pollution from livestock waste. *Resources, Conservation and Recycling* **2021**, *164*, 105199.
